# The molecular landscape of neurological disorders: insights from single-cell RNA sequencing in neurology and neurosurgery

**DOI:** 10.1186/s40001-023-01504-w

**Published:** 2023-11-16

**Authors:** Wireko Andrew Awuah, Arjun Ahluwalia, Shankaneel Ghosh, Sakshi Roy, Joecelyn Kirani Tan, Favour Tope Adebusoye, Tomas Ferreira, Hareesha Rishab Bharadwaj, Vallabh Shet, Mrinmoy Kundu, Amanda Leong Weng Yee, Toufik Abdul-Rahman, Oday Atallah

**Affiliations:** 1 Faculty of Medicine, Sumy State University, Zamonstanksya 7, Sumy, 40007 Ukraine; 2 School of Medicine, Queen’s University Belfast, Belfast, UK; 3 Institute of Medical Sciences and SUM Hospital, Bhubaneswar, India; 4 Faculty of Medicine, University of St Andrews, St Andrews, Scotland, UK; 5 Department of Clinical Neurosciences, School of Clinical Medicine, University of Cambridge, Cambridge, UK; 6 Faculty of Biology, Medicine and Health, The University of Manchester, Manchester, UK; 7 Faculty of Medicine, Bangalore Medical College and Research Institute, Bangalore, Karnataka India; 8 Faculty of Medicine, University of Malaya, Kuala Lumpur, Malaysia; 9 Department of Neurosurgery, Hannover Medical School, Carl-Neuberg-Strasse 1, 30625 Hannover, Germany

**Keywords:** Single-cell RNA sequencing, Brain tumours, Neurodegenerative disorders, Epilepsy and seizure disorders, Spinal cord diseases, Cerebrovascular diseases, Neurology, Neurosurgery

## Abstract

Single-cell ribonucleic acid sequencing (scRNA-seq) has emerged as a transformative technology in neurological and neurosurgical research, revolutionising our comprehension of complex neurological disorders. In brain tumours, scRNA-seq has provided valuable insights into cancer heterogeneity, the tumour microenvironment, treatment resistance, and invasion patterns. It has also elucidated the brain tri-lineage cancer hierarchy and addressed limitations of current models. Neurodegenerative diseases such as Alzheimer’s disease, Parkinson’s disease, and amyotrophic lateral sclerosis have been molecularly subtyped, dysregulated pathways have been identified, and potential therapeutic targets have been revealed using scRNA-seq. In epilepsy, scRNA-seq has explored the cellular and molecular heterogeneity underlying the condition, uncovering unique glial subpopulations and dysregulation of the immune system. ScRNA-seq has characterised distinct cellular constituents and responses to spinal cord injury in spinal cord diseases, as well as provided molecular signatures of various cell types and identified interactions involved in vascular remodelling. Furthermore, scRNA-seq has shed light on the molecular complexities of cerebrovascular diseases, such as stroke, providing insights into specific genes, cell-specific expression patterns, and potential therapeutic interventions. This review highlights the potential of scRNA-seq in guiding precision medicine approaches, identifying clinical biomarkers, and facilitating therapeutic discovery. However, challenges related to data analysis, standardisation, sample acquisition, scalability, and cost-effectiveness need to be addressed. Despite these challenges, scRNA-seq has the potential to transform clinical practice in neurological and neurosurgical research by providing personalised insights and improving patient outcomes.

## Background

Neurological disorders present significant challenges, often resulting in debilitating conditions that markedly degrade the quality of life. Recent advances in molecular biology and genomic technologies have significantly improved our understanding of these disorders, paving the way for more precise diagnostic and therapeutic strategies. Single-cell ribonucleic acid sequencing (scRNA-seq) has emerged as a particularly powerful tool, enabling the understanding of cellular heterogeneity and gene expression profiles at an unprecedented resolution [1].

The seminal work of Tang et al. [2] significantly contributed to both the conceptual framework and technical application of scRNA-seq. Their study marked a pivotal milestone by sequencing the transcriptome of individual mouse blastomeres and oocytes. One of the primary motivations behind pursuing scRNA-seq lies in its ability to present an in-depth profile of ribonucleic acid (RNA) molecules within individual cells, contrasting the traditional bulk RNA sequencing methods that analyse averaged gene expression profiles across larger cell populations. The precision of scRNA-seq provides superior resolution, capturing the entire genomic landscape within tissue and enabling more personalised treatment paradigms.

**Fig. 1:**
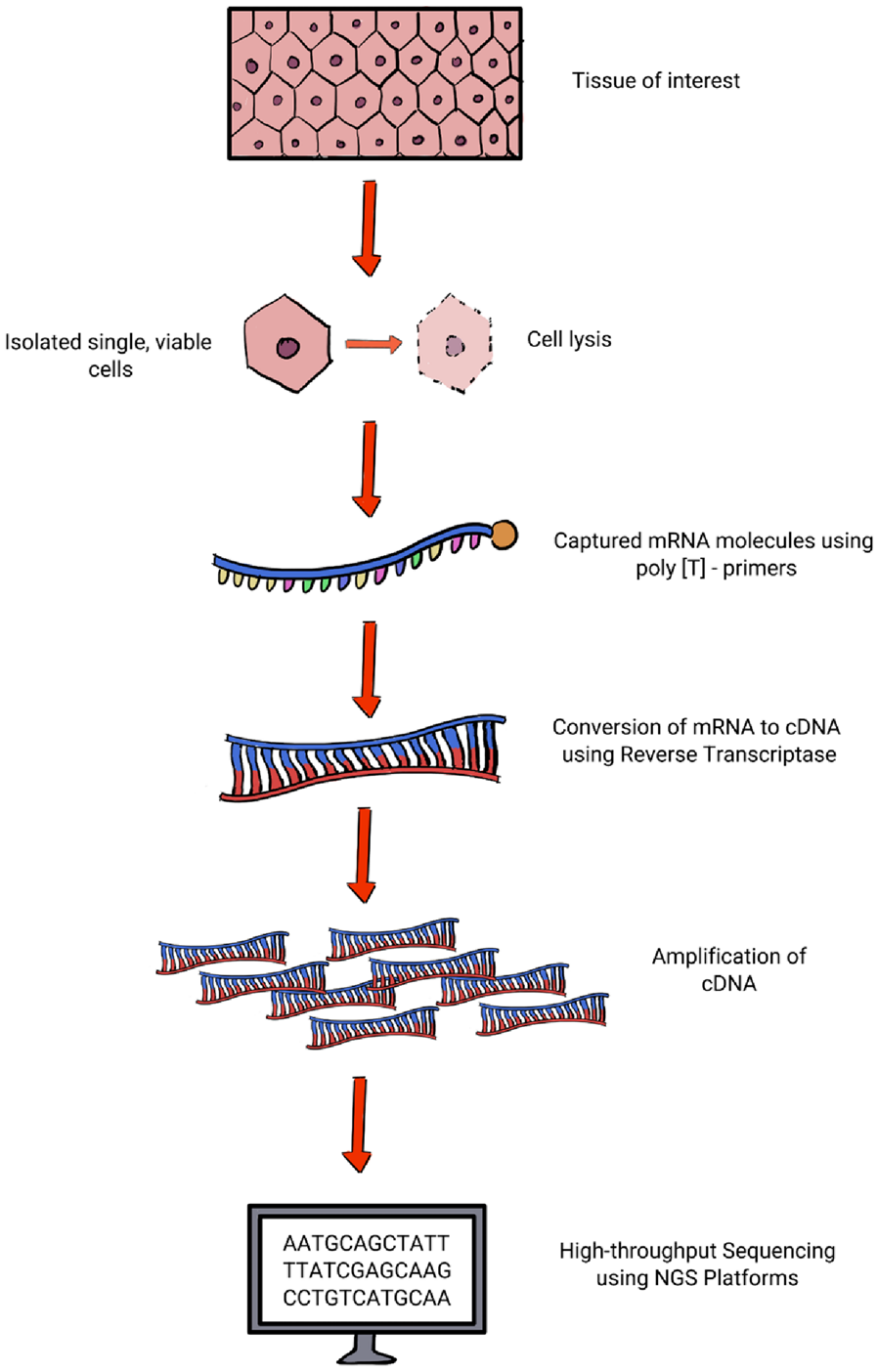
The general process of conducting single-cell RNA sequencing experiments. (Created with Krita.org). *mRNA* messenger ribonucleic acid, *cDNA* complementary deoxyribonucleic acid, *NGS* next generation sequencing

ScRNA-seq enables precise gene expression analysis at the individual cell level. Cells are extracted from tissue samples with isolation techniques. To capture RNA molecules, cells undergo lysis, during which poly[T]-primers selectively bind to polyadenylated messenger ribonucleic acid (mRNA) while excluding ribosomal RNAs. Subsequently, reverse transcriptase converts the captured mRNA into complementary deoxyribonucleic acid (cDNA). During this process, additional primers are introduced to enable detection, incorporate unique molecular identifiers (UMIs), and preserve cellular origin information [3]. The limited quantities of cDNA are then amplified, typically through polymerase chain reaction (PCR) or in vitro transcription, occasionally incorporating nucleotide barcode tagging to preserve cellular origin information. Each cell’s amplified and tagged cDNA is pooled and subjected to high-throughput sequencing using library preparation techniques and next-generation sequencing (NGS) platforms akin to bulk samples [3]. Figure 1 summarises the general process of conducting scRNA-seq experiments.

ScRNA-seq represents a rapidly evolving domain within transcriptomics, producing a vast amount of gene expression data at the single-cell (SC) level [4]. The application of scRNA-seq in neurological and neurosurgical research has grown notably, highlighting the heterogeneity of cell populations in the brain and its implications in brain tumours, neurodegenerative diseases (NDs), epileptic disorders, spinal conditions, and cerebrovascular diseases (CVDs) [5–8].

However, the use of scRNA-seq technology is not without challenges and limitations, including the high dimensionality, sparsity, noise, and scale of scRNA-seq data [9]. Moreover, cost, throughput, and sensitivity limitations potentially impact the accuracy and reproducibility of findings [4]. Addressing these challenges requires the development of improved statistical approaches and tools for informative data extraction [4].

By providing a comprehensive and high-resolution assessment of cellular heterogeneity and gene expression patterns, scRNA-seq has the potential to radically transform our understanding of neurological disorders and guide precision medicine approaches. This study aims to provide a thorough overview of how scRNA-seq technology has improved patient outcomes and refined our comprehension of neurological and neurosurgical research and practise.

## Methodology

This narrative review provides a comprehensive framework for assessing the application of scRNA-seq within neurosurgery and neurology. The inclusion criteria for this review encompassed only full-text articles written in English from 2003 to 2023. This time period allowed me appropriate evaluation within the field’s practises alongside innovations that had changed over a sizable period. To ensure an exhaustive literature search, several databases were employed, including PubMed, EMBASE, Google Scholar, the Cochrane Library, and Scopus. Key terms such as “scRNA Sequencing” and “Single-cell sequencing” were utilised in all searches, accompanied by additional terms comprising “Brain tumours,”, “Spinal Cord tumours,”, “Neurodegenerative Disorders”, “Cerebrovascular Disorders”, “strokes”, and “epilepsy”.

Additional sources were identified to augment the search strategy through a manual search of references cited in recent reviews focused on specific diseases. Rigorous exclusion criteria were adopted, involving the exclusion of standalone abstracts, case reports, posters, and unpublished or non-peer-reviewed studies. By instituting these criteria, the review sought to ensure the inclusion of high-quality and reliable evidence.

As for the scope of the review, no predetermined limit was set on the number of studies to be included, a strategy designed to gather comprehensive knowledge on the subject matter. The review included a range of study designs, including descriptive studies, animal-model studies, cohort studies, and observational studies. Moreover, it encompassed investigations conducted in both pre-clinical and clinical settings, offering a broad perspective on the use of scRNA-seq in neurosurgery and neurology research. A summary of the methodology employed is presented in Table 1.

## Results and discussion

### Applications of ScRNA-seq in neurological and neurosurgical diseases

#### Brain tumours

##### Gliomas

The application of scRNA-seq in neuro-oncological research has yielded valuable insights into previously unknown mechanisms. For instance, scRNA-seq has played a pivotal role in investigating the transcriptome landscape and drug resistance mechanisms in recurrent glioblastoma multiforme (GBM), a highly aggressive brain tumour [5]. Analysis of recurrent GBM cells using scRNA-seq has revealed significant findings, including the overexpression of stemness and cell-cycle-related genes, decreased microglial proportions, high expression of vascular endothelial growth factor A (VEGF-A), increased blood–brain barrier permeability, and activation of the *O*6-methylguanine deoxyribonucleic acid (DNA) methyltransferase-related pathway [5]. These findings elucidate tumour heterogeneity, the microenvironment, and potential therapeutic strategies for recurrent GBMs.

In addition, scRNA-seq and lineage hierarchy examination have revealed a neural tri-lineage cancer hierarchy centred around glial progenitor-like cells [10]. The progenitor population, abundant in cycling cells, serves as the primary source of other cell types within the tumour. This discovery creates opportunities for the targeting of progenitor cancer stem cells and exploration of innovative treatment approaches [10]. Moreover, the integration of scRNA-seq and multi-sector biopsies enables detailed cell classification and analysis, providing insights into distinct invasion patterns across tumour sub-regions, transcriptomic features of the glioma core and periphery, and chemokine/chemokine receptor interactions within the glioma microenvironment [11]. These advancements have contributed to our knowledge of cancer stem cells, tumour heterogeneity, and the aetiologies, progression, and treatment resistance of GBM.

ScRNA-seq has also addressed the limitations of current models by highlighting the inherent variability in gene expression profiles within primary GBMs [12]. This approach has revealed diverse transcriptional programs associated with crucial aspects of tumour biology, including oncogenic signalling, proliferation, complement/immune response, and hypoxia [12]. In addition, scRNA-seq sheds information on possible stemness regulators in the neoplasm microenvironment and emphasises the prognostic significance of intratumoral heterogeneity.

The immune landscape of GBM has been elucidated through the integration of scRNA-seq and flow cytometry, leading to the identification of longitudinal changes in immune cell composition during GBM progression [13]. The study identified the presence of proinflammatory microglia in developing GBMs, while end-stage tumours exhibited an abundance of anti-inflammatory macrophages and protumorigenic myeloid-derived suppressor cells. The impact of treatment responses was also explored, demonstrating the effects of temozolomide and irradiation on immune cell populations [13]. In addition, scRNA-seq analysis of GBM tissues has revealed substantial intratumoral heterogeneity and changes in the tumour microenvironment in recurrent GBMs compared to primary GBMs [5].

**Fig. 2:**
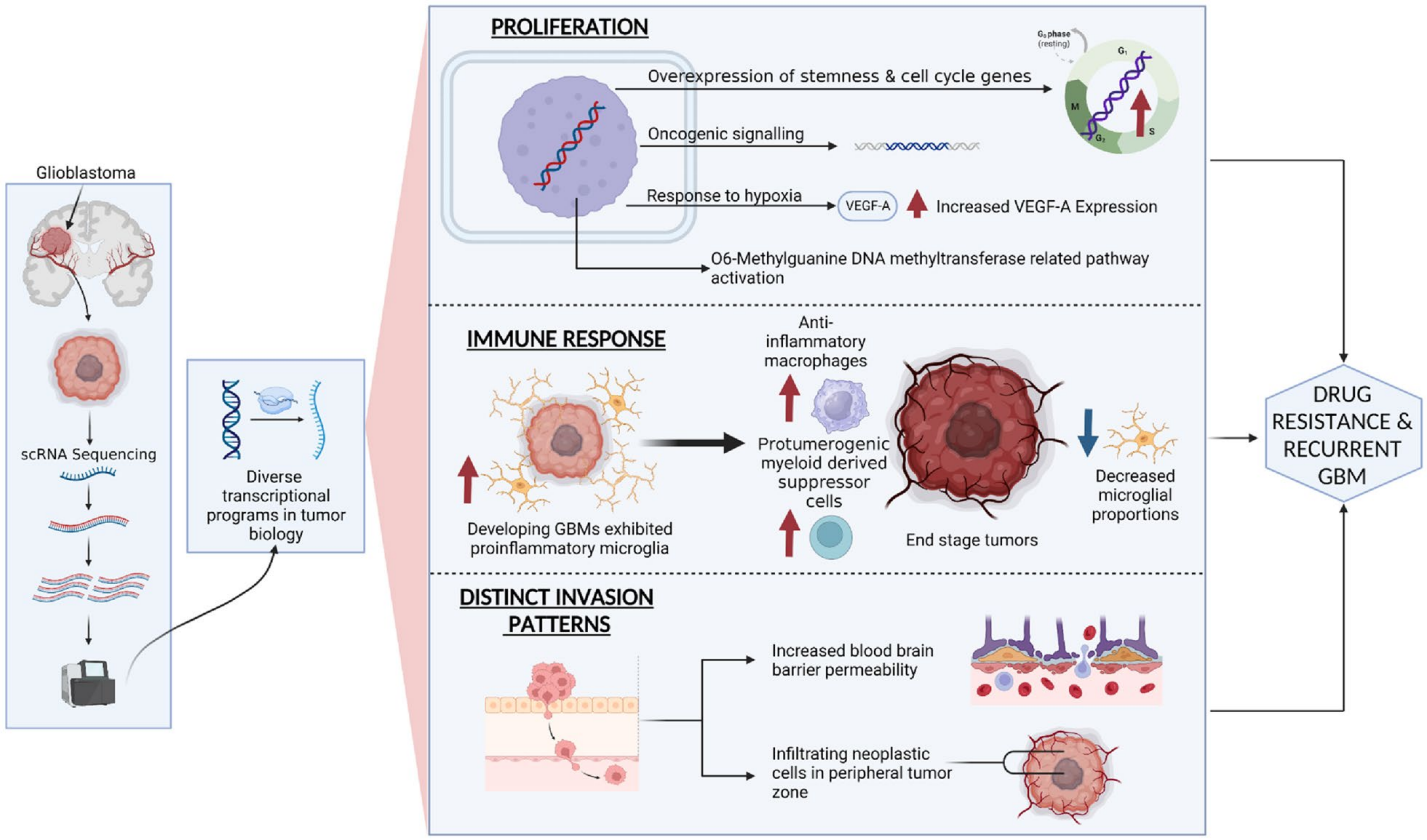
The utility of single-cell RNA sequencing in understanding tumour heterogeneity in glioblastoma multiforme. *scRNA-seq* single-cell ribonucleic acid sequencing, *GBM* glioblastoma multiforme, *VEGF-A* vascular endothelial growth factor-A, *DNA* deoxyribonucleic acid, *G*_*0*_* Phase* Gap 0 Phase, *G*_*1*_ Gap 1, *S* synthesis, *G*_*2*_ Gap 2, *M* mitosis

The use of scRNA-seq has also facilitated investigations into the impact of chromosomal instability (CIN) on gene expression and intra-tumour heterogeneity in GBM cancer stem cells (CSCs) [14]. The analysis revealed a correlation between gene expression and whole chromosome copy number in chromosomally unstable CSCs, highlighting the influence of CIN on transcriptional heterogeneity. GBM CSCs demonstrated significant expression diversity within pathways associated with stemness, lineage specification, and DNA damage response, underscoring the contribution of intra-tumour heterogeneity to GBM pathogenesis [14]. These findings provide a foundation for further exploration of GBM CSCs and potential therapeutic targets. The summarisation of the use of scRNA-seq in understanding the tumour heterogeneity of GBM is presented in Fig. 2.

ScRNA-seq analysis of the GBM tumour core and surrounding peripheral tissue has revealed significant cellular variation in the tumour’s genome and transcriptome [15]. Infiltrating neoplastic cells in peripheral regions have been identified, elucidating tumour invasion mechanisms. In addition, scRNA-seq has unveiled distinct molecular profiles and functional phenotypes of myeloid subpopulations within gliomas, including microglia, infiltrating monocytes and macrophages, and CNS border-associated macrophages [15]. Furthermore, sex-specific gene expression in glioma-activated microglia has been implicated in glioma incidence and outcomes [16].

Another notable discovery facilitated by scRNA-seq is the identification of cancer-associated fibroblasts (CAFs) in GBM [17]. Transcriptomic analysis of isolated cells with CAF characteristics from GBM specimens has revealed their distinct profiles and close association with other cell types, such as mesenchymal glioblastoma stem cells, endothelial cells, and M2 macrophages [17]. Moreover, CAFs have been found to induce M2 macrophage polarisation and enhance tumour growth in vivo. Furthermore, scRNA-seq and bulk RNA-seq databases have been used to construct a prognostic model for GBM survival prediction, incorporating differentially expressed genes associated with overall survival [18].

##### Meningiomas

ScRNA-seq has emerged as a valuable tool for investigating the underlying mechanisms of malignancy and recurrence in high-grade meningiomas, which often exhibit poor treatment outcomes [19]. Through scRNA-seq analysis, distinct subpopulations of initiating cells characterised by oestrogen sulfotransferase family 1E member 1 (SULT1E1) expression have been identified in high-grade meningiomas. These cells play a critical role in promoting tumour progression and recurrence by modulating M2-type macrophage polarisation. Researchers have further explored these cells using a patient-derived meningioma organoid (MO) model, which accurately replicates the aggressiveness of SULT1E1+ cells. Notably, these MOs exhibit invasive behaviour in the brain after orthotopic transplantation. Targeted interventions in the MO model have demonstrated the effectiveness of the synthetic compound SRT1720 in targeting SULT1E1+ cells, suggesting its potential as a systemic treatment and radiation sensitiser [19].

In addition to studying tumour cells, scRNA-seq analysis of human dura and primary meningioma samples has provided insights into the immune microenvironment within the dura, the protective membrane surrounding the brain [20]. The analysis has revealed individual immune responses in the dura compared to meningiomas, suggesting distinct immune characteristics of the dura. Furthermore, the study highlighted the diverse and heterogeneous nature of endothelial cells and fibroblasts within the dura. By examining the spatial relationship between immune cell types and vasculature in non-tumour-associated dura, researchers gained insights into the organisation of these cells. Notably, there was a significant overlap in T-cell receptor sequences between dura and meningioma samples, indicating a potential connection between the immune response in the dura and the development of meningiomas. In addition, the presence of copy number variant heterogeneity within meningioma samples suggests genetic diversity that may influence tumour progression. Thus, scRNA-seq has improved our understanding of the human dura and meningioma at the single-cell level, providing novel insights into the roles of the dura in immune surveillance and its interactions with the tumour microenvironment [20].

Moreover, integrating scRNA-seq with various genomic, transcriptomic, and proteomic approaches can address the limited understanding of meningioma biology and the lack of adequate medical therapies. A detailed analysis has identified distinct DNA methylation groups associated with unique clinical outcomes, biological drivers, and therapeutic vulnerabilities [21]. Meningiomas with an intact expression of the Merlin protein (Neurofibromin 2/Merlin gene) exhibited the most favourable outcomes and were susceptible to cytotoxic therapy. Immune-enriched meningiomas displayed intermediate outcomes and were characterised by immune infiltration, human leukocyte antigen (HLA) expression, and lymphatic vessels. Conversely, hypermitotic meningiomas had the worst outcomes and were driven by genetic and epigenetic mechanisms that promote cell cycle progression and resistance to cytotoxic therapy. Cytostatic cell cycle inhibitors have shown efficacy in attenuating meningioma growth in experimental models and patients, providing potential therapeutic targets to improve treatment options [21].

**Fig. 3:**
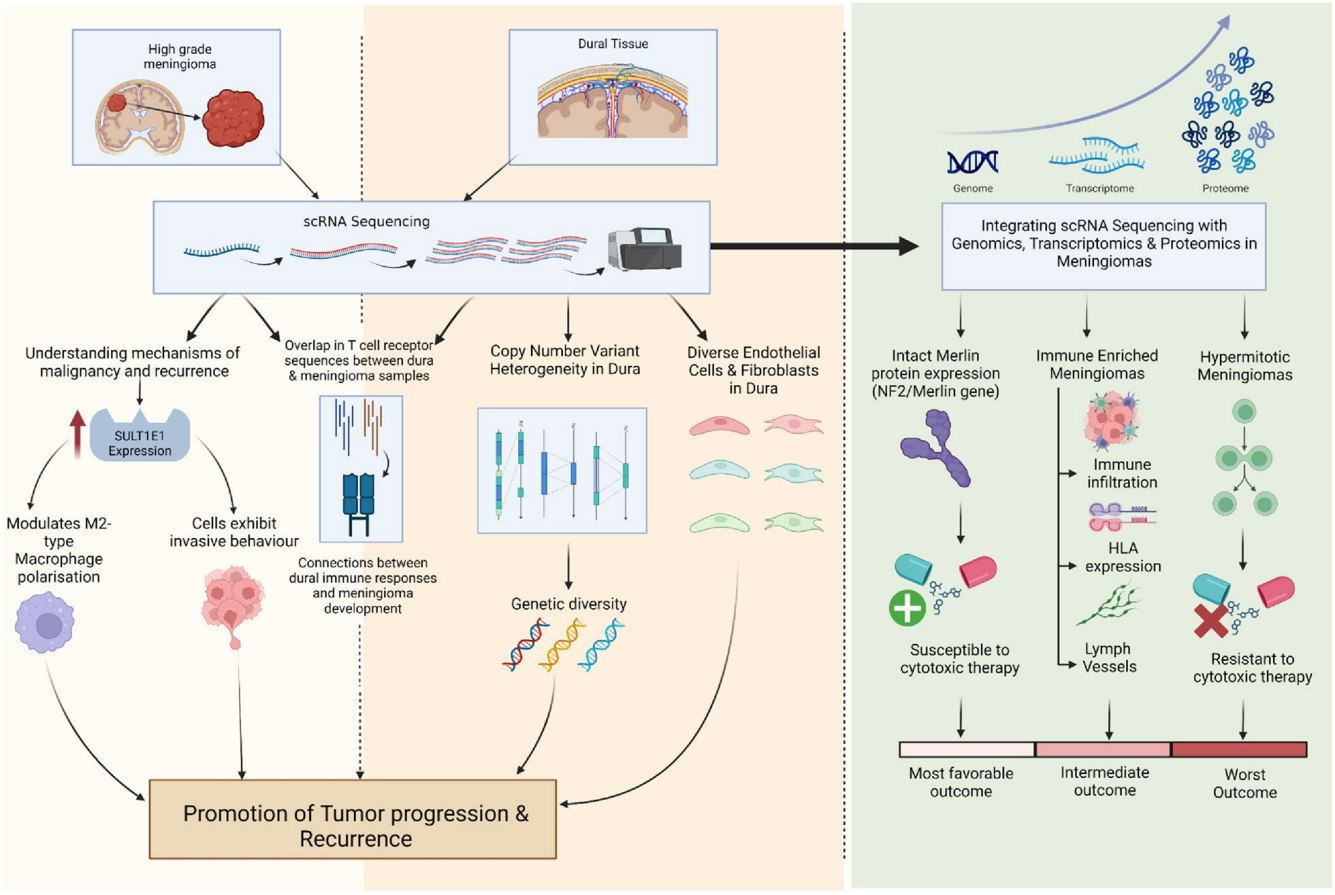
The utility of single-cell ribonucleic acid sequencing in predicting tumour progression and recurrence, as well as clinical outcome in meningiomas. *scRNA* sequencing, single-cell ribonucleic acid sequencing, *HLA* human leukocyte antigen, *NF2* neurofibromin 2, *SULT1E1* sulfotransferase family 1E member 1

Furthermore, scRNA-seq has shed light on regional alterations in chromosome structure underlying clonal transcriptomic, epigenomic, and histopathologic signatures within meningiomas [20]. Researchers have used multiplatform molecular profiling of spatially distinct samples and integrated sample coordinates with preoperative magnetic resonance images to identify specific regions enriched with proliferating cells exhibiting a high apparent diffusion coefficient [22]. These regions are associated with developmental gene expression programs. A human cerebral organoid meningioma model has been developed to elucidate the functional significance of these findings. Through scRNA-seq, live imaging, Clustered Regularly Interspaced Short Palindromic Repeats (CRISPR) interference, and pharmacological experiments, Cadherin 2 (CDH2) and Receptor-Type Tyrosine–Protein Phosphatase Zeta (PTPRZ1) have been validated as potential therapeutic targets based on their identification as high anti-drug conjugate (ADC) marker genes [20]. Figure 3 summarises the utility of scRNA-seq in understanding meningioma tumour heterogeneity.

#### Neurodegenerative diseases and ScRNA-seq

ScRNA-seq has emerged as a powerful tool in the study of NDs, including Alzheimer’s disease (AD), Parkinson’s disease (PD), amyotrophic lateral sclerosis (ALS) and Huntington’s disease (HD). This technology enables the investigation of molecular changes at the single-cell level, revealing cellular heterogeneity in affected brain regions [7]. By applying scRNA-seq, researchers have made significant contributions to understanding various NDs, by uncovering specific cell types, their states, and dysregulation underlying these conditions [23, 24].

In the case of AD, scRNA-seq has been instrumental in the molecular subtyping of the disease and identifying novel mechanisms and therapeutic targets. Gene expression profiles at the single-cell level have identified three major molecular subtypes of AD, each associated with distinct dysregulated pathways involved in tau-mediated neurodegeneration, amyloid-β neuroinflammation, synaptic signalling, immune activity, mitochondrial organisation, and myelination [23]. Moreover, scRNA-seq has provided valuable insights into microglia, the resident immune cells of the CNS**,** in AD brains, revealing functional heterogeneity and potential targets for therapeutic intervention [5]. Transcriptomic changes in microglia during severe neurodegeneration have been explored using scRNA-seq, uncovering distinct reactive microglia phenotypes and disease-stage-specific microglia cell states [9]. In addition, single cell analysis has helped explore the genetic landscape of the frontal cortex afflicted by AD. Various dysregulated genes associated with AD were found to be common and upregulated in various cells, especially astrocytes [25]. Conversely, genes associated with synaptic transmission and RNA splicing were downregulated [25].

Similarly, in PD, scRNA-seq has aided in identifying new therapeutic targets and improving our understanding of the disease’s molecular mechanisms. Analysis of PD risk variants has revealed cell-type-specific gene expression patterns in glia and neurons, highlighting the role of astrocytes and microglia in unfolded protein response and cytokine signalling [26]. ScRNA-seq has also played a critical role in analysing human induced pluripotent stem cell (iPSC)-derived dopaminergic neurons, providing insights into PD pathogenesis and potential cell replacement therapies [27, 28]. Furthermore, scRNA-seq has contributed to exploring cellular heterogeneity, uncovering mechanisms, and identifying therapeutic targets for PD by analysing midbrain specimens from PD patients and healthy individuals [29]. Analysis of samples from PD patients also revealed that CD4+ CTLs are more prevalent and have an increased response to interferon-gamma, signalling enhanced cell attachment. Notably, endothelial cells (ECs) in the PD patient’s brain are especially sensitive to IFNG compared to other brain cells [30]. It was also revealed that CD4+ T cells might enter the CNS by interacting with specific pairs of proteins, particularly through areas, where brain–blood barriers might be weaker [30].

In the case of ALS, scRNA-seq analysis of motor neurons derived from ALS patients has uncovered disease-specific gene expression changes and disrupted cellular pathways associated with ALS pathogenesis [31]. Furthermore, analysis of CSF samples from patients with ALS also reveals a high number of cytotoxic T cells with a lower number of monocytes and normal T cells when compared to samples taken from healthier patients. Moreover, there was a dysregulation of genes controlling cellular toxicity [32]. Further sequencing coupled with immune profiling allowed the identification of novel CD8+ T-cell subtypes with varied stemness gene expression, possessing signature genes of both monocytes and terminal effector T cells, potentially pivotal in ALS pathogenesis [33].

A recent HD research also generated human striatal organoids, verified by immunohistochemistry and scRNA-seq [34]. Sequencing revealed increased HSF1 colocalization in mitochondria and striatal organoids, suggesting mHSF1’s potential as a therapeutic target [34]. Furthermore, analysis through scRNA-seq has delineated the differential gene expression within the striatal cell population, which has enabled the identification of specific genes that are upregulated in HD compared to other NDs, such as AD [35].

The diverse applications of scRNA-seq in studying NDs, including AD, PD, and ALS, have revolutionised our understanding of these diseases by uncovering cellular susceptibilities and molecular mechanisms underlying these conditions. This technology identifies distinct cell types and states, offering new perspectives on the pathogenesis of NDs, and provides insights into potential biomarkers and therapeutic targets. However, analysing the vast amount of data generated by scRNA-seq presents challenges, requiring the development of computational and analytical tools for comprehensive interpretation [24]. The studies discussed in this context highlight the significant contributions of scRNA-seq in unravelling the cellular and molecular complexities of NDs and paving the way for future advancements in diagnosis and treatment strategies.

**Fig. 4:**
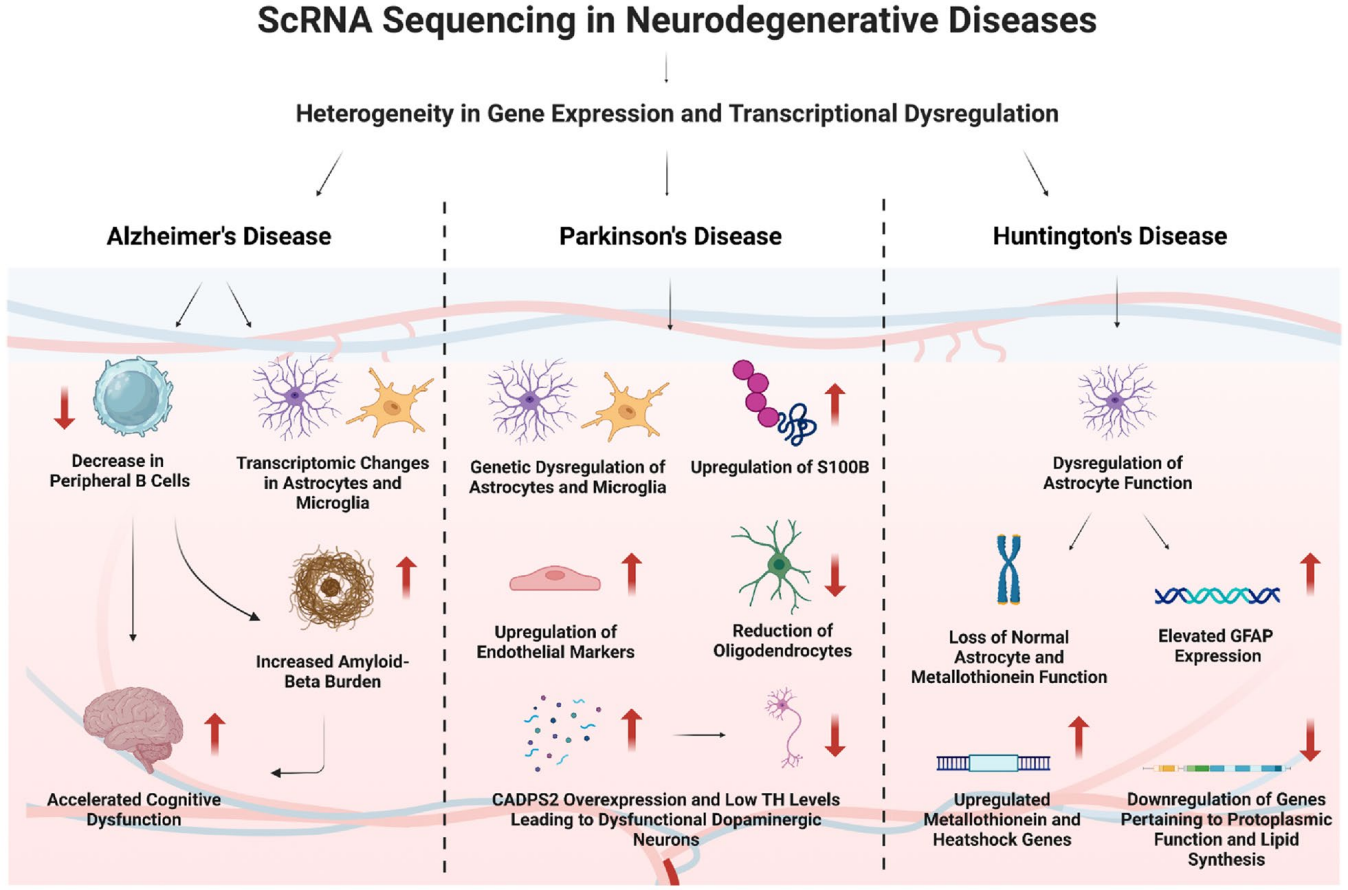
Uncovering cellular heterogeneity in neurodegenerative diseases using single-cell RNA sequencing (created with Biorender.com). *ScRNA* sequencing, single-cell ribonucleic acid sequencing, *GFAP* glial fibrillary acidic protein, *CADPS2* calcium-dependent secretion activator 2, *TH* tyrosine hydroxylase

The uncovering cellular heterogeneity in NDs using scRNA sequencing is illustrated in Fig. 4.

#### Epilepsy, seizure disorders and ScRNA-seq

The rise of new molecular mapping techniques has facilitated the emergence of scRNA-seq as a transformative technology in epilepsy research. This powerful tool enables researchers to investigate the cellular and molecular heterogeneity that underlies epilepsy, which is characterised by dysregulated gene expression patterns leading to altered neuronal excitability, synaptic plasticity, and network dysfunction.

One area where scRNA-seq has proven particularly valuable is in understanding the complex cellular and molecular changes associated with epilepsy. By applying nuclear RNA-seq to isolate specific cell populations from human neocortex samples associated with temporal lobe epilepsy (TLE), researchers have identified a subpopulation of glial cells expressing characteristics of both reactive astrocytes and oligodendrocyte precursor cells (OPCs). This mixed glia, marked by GFAP and Oligodendrocyte transcription factor 2 (OLIG2) markers’ expression, was found exclusively in TLE [36].

In addition to investigating cellular heterogeneity, scRNA-seq has been employed to explore the multifaceted nature of epileptogenic triggers. Researchers have used this technique to study microglia obtained from patients with drug-refractory epilepsy (DRE) and have discovered higher levels of pro-inflammatory cytokine markers, including interleukin-1 beta (IL1B), within microglia from DRE patients compared to those from brain samples without pathologies [37]. This finding suggests a potential link between dysregulated pro-inflammatory mechanisms and the development of DRE, offering insights into potential therapeutic interventions. Another trigger investigated in epilepsy is ion dysregulation resulting from mutations or misexpression of ion channels. A study focused on seizures induced by gliomas, a type of brain tumour, used scRNA sequencing techniques to examine the transcriptome of cycling progenitor cells obtained from human glioma samples [38]. The study revealed the downregulation of potassium channels attributed to the interaction between immunoglobulin superfamily member 3 (IGSF3) and inwardly rectifying potassium Kir4.1, suggesting impaired potassium buffering mechanisms as a potential mechanism underlying epileptogenesis in the context of gliomas.

To address the network dysfunction observed in epilepsy, researchers have employed differential RNA sequencing analysis to isolate nuclei enriched with neurons, astrocytes, and OPCs from human neocortex samples [39]. This approach has identified dysregulated pathways in these cell types, including an immature phenotype switch in TLE astrocytes and a mixed population of glial cells expressing both astrocyte and OPC-like progenitor markers in TLE.

Exploring the role of immune system dysregulation in epilepsy, scRNA-seq and T-cell receptor sequencing have been used to investigate the innate and adaptive immune responses in focal epilepsy. Analysis of peripheral blood mononuclear cells (PBMCs) from epilepsy patients revealed differences in the composition of adaptive and innate immune cells between poorly controlled (PC) and well-controlled (WC) subjects [40]. PC epilepsy subjects showed an elevated proportion of memory CD4+ and CD8+ T-cells and cytotoxic cytokine-expressing Natural Killer T cells, while CD14+ and CD16+ monocytes and B memory cells were depleted. Ligand/receptor network analyses highlighted disrupted immune homeostasis in poorly controlled focal epilepsy.

**Fig. 5:**
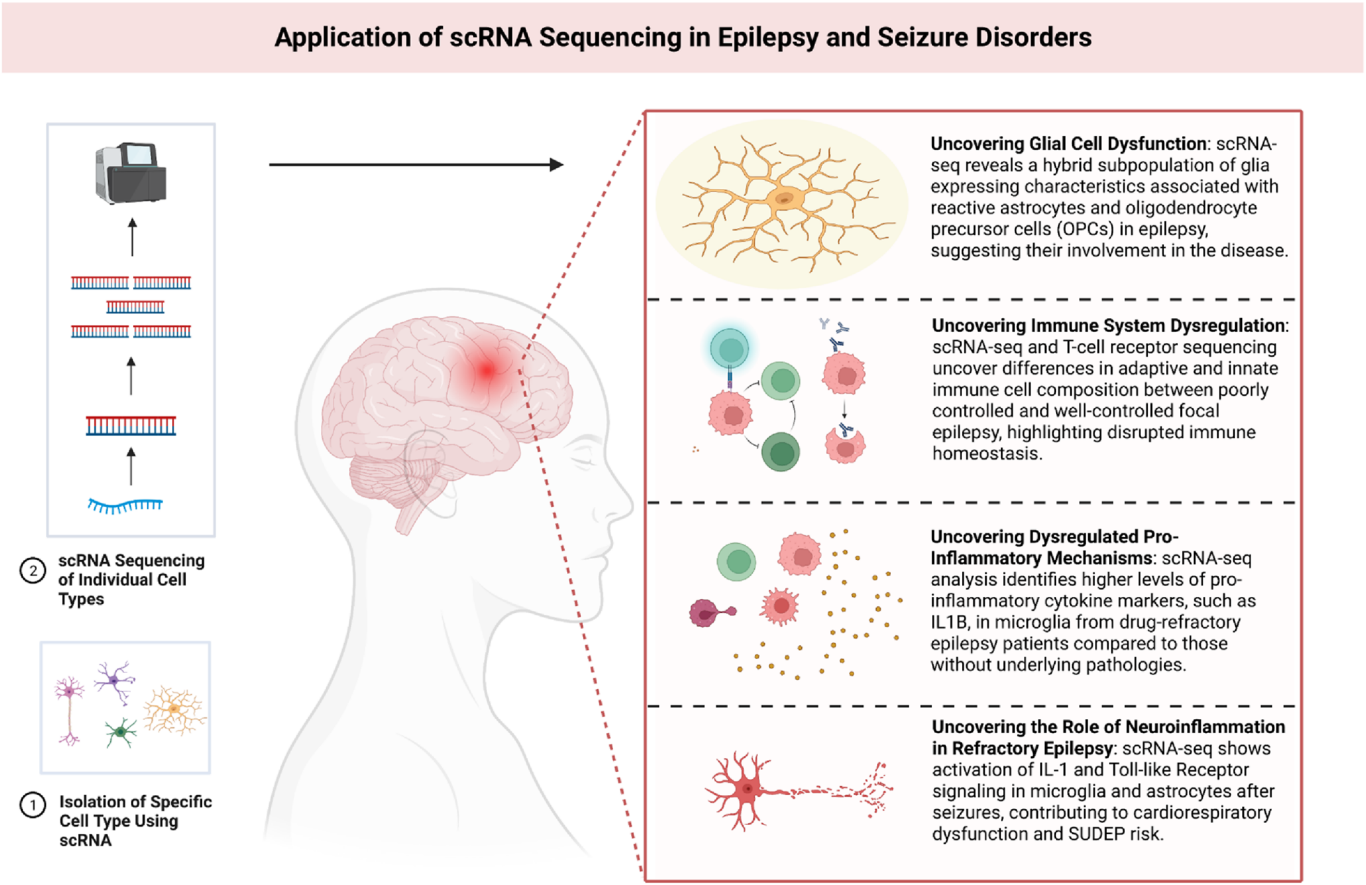
Understanding disease pathways and processes in epilepsy and seizure disorders using single-cell RNA sequencing. (using Biorender.com). *scRNA-seq* single-cell RNA sequencing, *IL-1* interleukin 1, *SUDEP* sudden unexpected death in epilepsy, *OPC* oligodendrocyte progenitor cells, *IL1B* interleukin-1 beta

Furthermore, investigations into neuroinflammation mediated by microglia and astrocytes in patients with refractory epilepsy have revealed the activation of specific inflammatory pathways, such as Interleukin 1 (IL-1) and Toll-like Receptor signalling, following repeated seizures [41]. Cytokine–chemokine array analysis confirmed the presence of neuroinflammation in brain regions involved in cardiorespiratory control, contributing to our understanding of the consequences of uncontrolled seizures and identifying potential therapeutic targets to improve cardiorespiratory function and reduce the risk of sudden unexpected death in epilepsy (SUDEP) [41]. The application of scRNA-seq in epilepsy and seizure disorders are illustrated in Fig. 5.

#### Spinal cord diseases and ScRNA-seq

##### ScRNA-seq and spinal cord injuries (SCIs)

SCIs are complex pathological conditions involving diverse cellular constituents and intricate signalling mechanisms. By leveraging scRNA-seq, researchers have made significant strides in unravelling the cellular dynamics underlying SCIs. This technique identifies and characterises distinct cell types, including neurons, astrocytes, microglia, oligodendrocytes, endothelial cells, and fibroblasts, shedding light on their transcriptional landscapes and uncovering previously unexplored diversity and adaptability.

Through scRNA-seq analysis of spinal lesion tissues at various timepoints after SCIs, researchers have identified crucial cell populations involved in angiogenesis, such as vascular endothelial cells [42]. Microglia and macrophages were found to modulate specific subsets of endothelial cells through signalling pathways involving Secreted Phosphoprotein 1 (SPP1) and Insulin-Like Growth Factor 1 (IGF1), promoting angiogenesis [43]. In addition, scRNA-seq revealed a novel microglial subtype, similar to disease-associated microglia (DAM) observed in other contexts, which contributed to hindlimb locomotor function recovery following injury [44]. Furthermore, B cells displayed diverse developmental states. They became more prominent during the chronic stages of SCIs, suggesting their significance in the immune response at the injury site [45]. Recent analysis of murine models has also indicated a permanent two-wave activation of microglia 14 day post-injury [46]. This mode of sequencing extends its utility further to decipher the intricate landscape of gene expression alterations in the immediate phases of SCI, encompassing both long non-coding RNAs and messenger RNAs, providing valuable insights into the molecular responses to injury while elucidating potential targets for intervention [47].

Recent advances in scRNA-seq have profoundly enhanced the understanding of regenerative strategies for SCIs. Research utilising this technique has helped identify stress-responsive regenerating cells (SrRCs) essential for post-SCI neuronal regeneration [48]. This high-resolution sequencing technique has further elucidated the dynamics of neural stem cells (NSCs) and oligodendrocyte progenitor cells (OPCs) after SCIs. Specifically, scRNA-seq has unveiled distinct NSC subpopulations, revealing their activation patterns and transcriptional shifts in response to injury [49]. Similarly, OPCs demonstrated heightened activation and an augmented neurogenic capacity post-SCI [50]. These revelations offer vital insights into spinal cord repair processes and highlight potential therapeutic avenues for fostering axonal regeneration and functional recovery.

In addition, scRNA-seq has been pivotal in elucidating patterns of lower motor neuron (LMN) abnormalities, particularly in the context of nerve–muscle groups identified as potential recipients for nerve transfer surgery in individuals grappling with chronic SCI [51]. Finally, it has underscored the potential benefits of a ketogenic diet, demonstrating its capacity to enhance the steroid anabolic pathway in rodent models afflicted with SCI, with promising implications for optimising the immune microenvironment and promoting myelin growth [52].

**Fig. 6:**
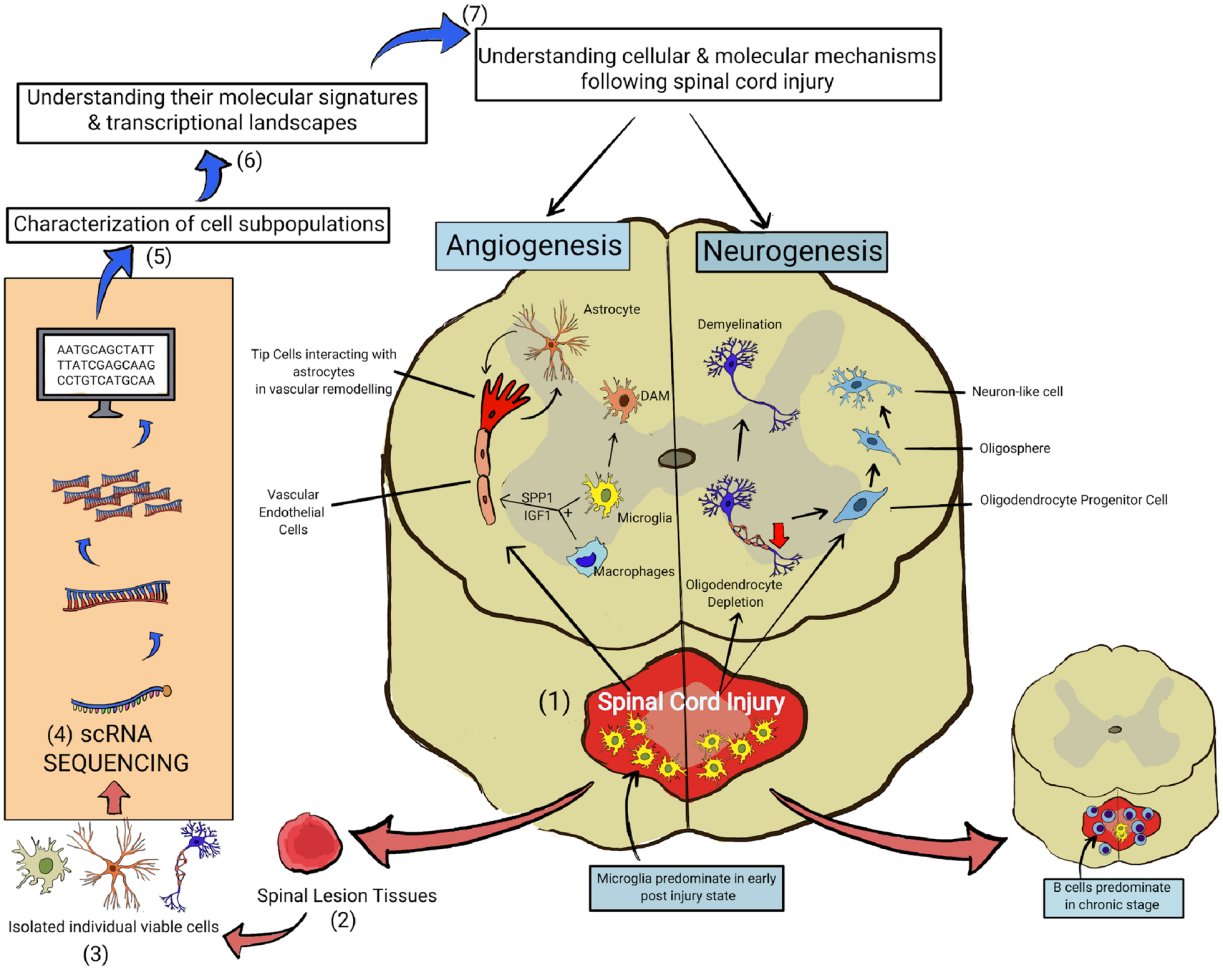
Insights into single-cell RNA sequencing in cellular dynamics and molecular responses following spinal cord injury. (Created with Krita.org). *ScRNA-seq* single-cell RNA sequencing, *DAM* disease associated microglia, *SPP1* secreted phosphoprotein 1, *IGF1* insulin-like growth factor 1

In sum, these collective investigations, leveraging the power of scRNA-seq, have significantly advanced our comprehension of SCI pathophysiology and have illuminated promising avenues for SCI intervention, through means of targeted angiogenic and neurogenic therapy. The insights into scRNA-seq in cellular dynamics and molecular responses following SCIs are illustrated in Fig. 6.

##### Spinal tumours and ScRNA-seq

ScRNA-seq has emerged as a powerful tool for studying various tumours’ molecular attributes and cellular heterogeneity, including those affecting the spinal cord. By examining gene expression patterns in individual cells, scRNA-seq enables comprehensive insights into the cellular diversity and molecular subtypes of spinal cord tumours.

In the case of spinal ependymomas, scRNA-seq analysis has unveiled distinct functional subsets of tumour-associated macrophages (TAMs) with different roles in immune responses, apoptosis induction, and tumour angiogenesis [53]. In addition, scRNA-seq, combined with SC Assay for Transposase-Accessible Chromatin using sequencing (ATAC-seq), has identified critical regulators of TAM functional heterogeneity [53]. The application of scRNA-seq has also facilitated the examination of intratumoral heterogeneity and the developmental origins of ependymomas across different molecular groups and anatomical locations [54]. This approach has refined our understanding of the ependymoma cellular hierarchy and provided insights into their aggressive characteristics, such as late recurrence and treatment resistance.

Furthermore, scRNA-seq has provided a comprehensive view of chordoma’s poorly understood tumour heterogeneity and potential therapeutic targets. Through scRNA-seq analysis, researchers identified distinct subclusters of chordoma cells exhibiting epithelial-like extracellular matrix characteristics, stem cell features, and immunosuppressive properties [55]. Immunosuppression was mainly observed in Regulatory T Cells (Tregs) and M2 macrophages, while the Transforming Growth Factor Beta (TGF-β) signalling pathway was implicated in tumour progression, invasion, and immunosuppression [55].

ScRNA-seq has emerged as a transformative technology, providing valuable insights into the complex cellular dynamics and molecular responses associated with SCIs and tumours. By enabling the identification of specific cell types, the characterization of their transcriptional landscapes, and the exploration of cellular interactions, scRNA-seq holds immense potential for identifying therapeutic targets and guiding future research in these fields.

#### Cerebrovascular diseases and ScRNA-seq

To unravel the intricate cellular dynamics and molecular mechanisms underlying stroke and cerebrovascular diseases (CVDs), groundbreaking research using scRNA-seq has emerged. These studies offer valuable insights into the role of specific genes, cell populations, and cellular plasticity, providing a comprehensive understanding of these complex conditions.

##### Stroke

ScRNA-seq has advanced our understanding of microglial biology, astrocytic responses, and immune cell dynamics in neurological disorders [56]. RNA expression patterns in microglia have identified distinct states and persistent inflammatory microglia enriched in chemokines, revealing molecular and functional diversity in age-related neuroinflammation. scRNA-seq in a mouse model of ischemic stroke unveiled cellular heterogeneity, distinct brain clusters, microglial subtypes, and trajectory branches of monocytes and macrophage subsets, offering drug discovery targets [57]. Astrocytic responses in stroke and ageing identified major subtypes, gene expression changes, and metabolic pathways, providing targets for neuroprotection [57]. scRNA-seq explored immune cell responses in the aged brain after stroke, revealing distinct microglial subsets and infiltrated myeloid cell subpopulations [58]. Significant changes were also seen in the populations of NK cells and CD14+ monocytes. NK cell numbers and activity rose, a finding further validated by flow cytometry, and their activity was also heightened [59]. This was coupled with a notable decrease of CD14+ monocytes, signifying the change in the immune landscape [59].

ScRNA-seq techniques advanced neurogenomics by investigating the molecular underpinnings of neurological disorders. Microglial states and persistent inflammatory microglia enriched in chemokines shed light on their diversity and involvement in neuroinflammation. Besides underscoring the diverse microglial subtypes as well as genetic expression post-ischaemic stroke, the utilisation of scRNA-seq has also unveiled information regarding the alteration in the cell communication landscape [60]. Post-stroke marks a shift in interactions from predominantly fibroblastic cells to mostly microglia and CNS-associated macrophages by means of chemokines, cytokines, and TNF families [60]. Furthermore, sequencing along with epigenetic analysis has allowed the identification of neurons that induce the expression of genes that can prevent further cell death and inflammation while stimulating peri-infarct neurons with gene expression patterns linked to brain injury recovery [61].

ScRNA-seq in stroke models uncovered cellular heterogeneity, gene expression patterns, and trajectory branches, suggesting drug discovery targets. Astrocytic responses revealed major subtypes, gene expression changes, and metabolic pathways as potential targets for neuroprotection [58]. Furthermore, scRNA-seq has enabled the discovery of an antioxidant enzyme, and revealed a unique stroke associated microglial, with the enzyme playing a pivotal role in their antioxidant defence and preventing further brain damage and cell death [62]. This novel technique explored immune cell responses post-stroke, identifying distinct microglial subsets and infiltrated myeloid cells, enhancing our understanding of immune dynamics [58].

##### Cerebral aneurysms

Cerebral aneurysms are localised dilations of cerebral arteries that pose a significant risk of rupture, leading to subarachnoid haemorrhage and potentially devastating neurological consequences. ScRNA-seq studies have been instrumental in unravelling the cellular and molecular heterogeneity within cerebral aneurysms. For instance, Wen et al. [63] employed scRNA-seq to identify distinct subpopulations of smooth muscle cells, endothelial cells, and inflammatory cells within aneurysm walls. The research underscored the role of inflammatory responses and extracellular matrix remodelling in aneurysm pathogenesis [63]. Furthermore, it was observed that upregulated genes in aneurysm-derived vascular smooth muscles are associated with arterial mineralization and atherosclerosis, with key genes, including PTX3, SPP1, and LOX [63]. In contrast, genes related to vasodilation and physiological regulation such as MGP, ACTA2, and MYL9 were predominantly enriched in conventional vascular smooth muscles [63].

ScRNA-seq has also aided in the discovery and analysis of the transcriptional signature linked to the metabolic pathways of ATP generation during aneurysms, with observations pointing to the process being downregulated during aneurysm formation and rupture [64]. Concurrently, this condition also resulted in a marked expansion of the total macrophage population, a trend that was accentuated upon rupture. Within this context, both inflammatory and resolution-phase macrophages were discerned, accompanied by a pronounced surge in neutrophils associated with aneurysm rupture. The model of single cell transcriptomics utilised for understanding novel gene signatures associated with aneurysm formation and rupture, supports the innovation of potential therapeutic intervention targets [64].

##### Intracranial haemorrhages (ICHs)

ICHs, such as intracerebral and subdural hematomas, are characterised by bleeding within the brain parenchyma or between the brain and its surrounding membranes, respectively. Although less explored using scRNA-seq compared to other cerebrovascular diseases, emerging studies have begun elucidating the cellular dynamics and gene expression changes associated with ICHs [65]. A recent study by Li et al. [58] used scRNA-seq to profile the immune cell landscape within ICHs lesions, revealing distinct subpopulations of microglia and infiltrating immune cells involved in neuroinflammation and tissue repair processes. These findings underscore the potential of scRNA-seq for uncovering novel therapeutic targets and developing personalised treatment strategies for ICHs.

Through scRNA-seq and spatial transcriptomics, researchers examined post-SAH alterations in meningeal lymphatic vessels, pinpointing thrombospondin 1 (THBS1) and S100A6 as crucial to SAH outcomes [66]. Ligand interactions were identified as regulators of meningeal lymphatic endothelial cell apoptosis via distinct signalling pathways [66]. Furthermore, scRNA-seq allowed the identification of a set of genes responsible for oligodendrocyte differentiation and fatty acid metabolism [67]. In addition, further investigation provided evidence of its capacity to reduce brain damage after ICH [67]. These findings underscore a promising therapeutic strategy through targeting various genes and signalling pathways, allowing more personalised treatments.

**Fig. 7:**
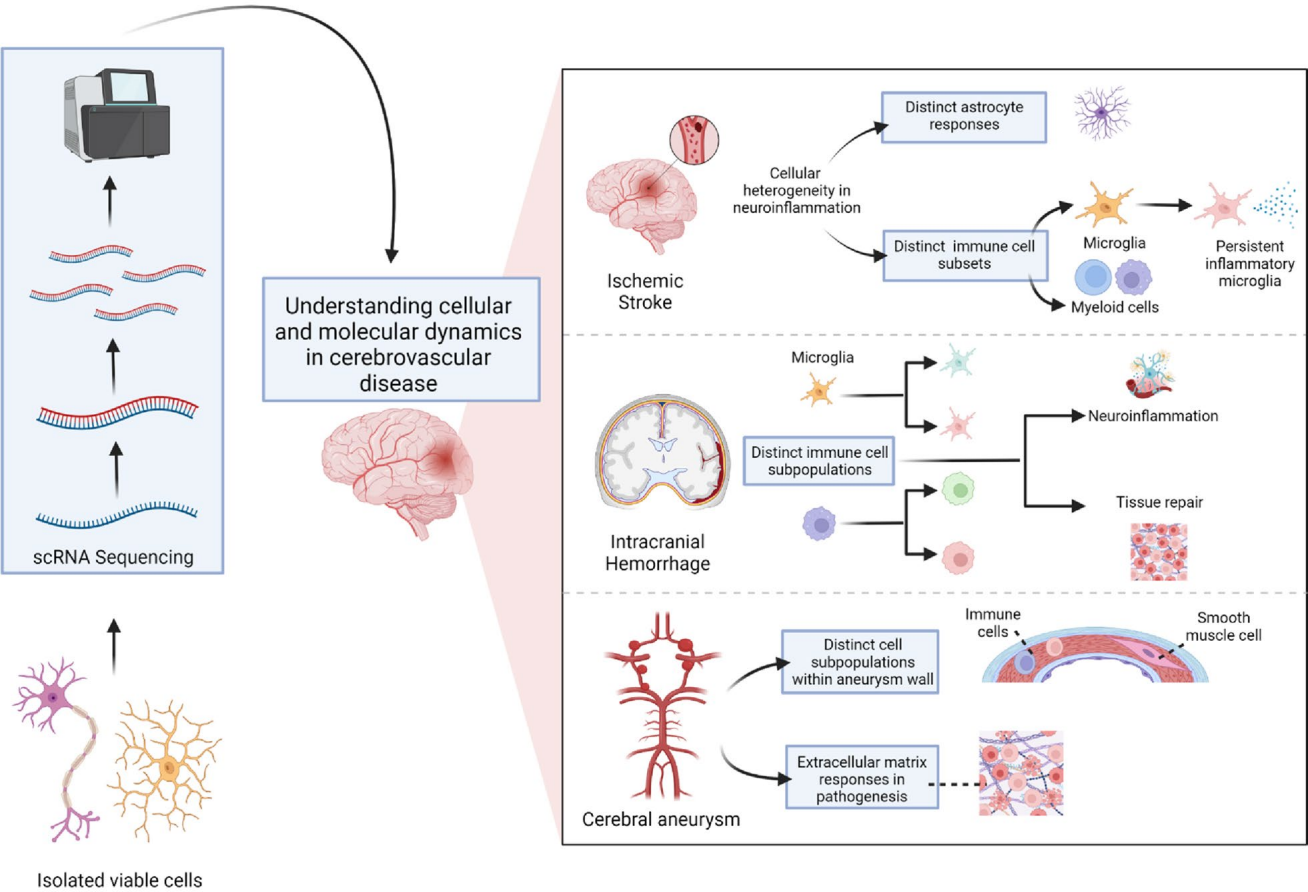
The cellular dynamics and molecular responses to cerebrovascular disorders using single-cell RNA sequencing. (Created with Biorender.com). *scRNA-seq* single-cell RNA sequencing

The insights into cellular dynamics and molecular responses to CVDs using scRNA sequencing are illustrated in Fig. 7.

### Clinical translation and practical consideration

ScRNA-seq has emerged as a powerful tool for understanding cellular heterogeneity and gene expression profiles at the SC level. In neurological and neurosurgical research, scRNA-seq holds immense potential to transform clinical practice and patient care. This section aims to explore the translational potential of scRNA-seq, discuss the practical considerations for clinical implementation, and highlight the role of scRNA-seq in clinical trials and therapeutic validation.

#### Translational potential of scRNA-seq

##### Clinical biomarkers

scRNA-seq offers a unique opportunity to identify novel biomarkers for neurosurgical diseases, enabling early diagnosis, prognosis assessment, and personalised treatment selection. By characterising gene expression patterns at the single-cell level, researchers can uncover molecular signatures associated with disease progression, treatment response, and therapeutic resistance [68]. For example, scRNA-seq studies have identified specific cell types or gene expression profiles associated with aggressive tumour subtypes or drug resistance in GBM [10, 20]. These findings have the potential to guide clinical decision-making and improve patient outcomes.

##### Precision medicine

The application of scRNA-seq in precision medicine approaches holds great promise for personalised therapies in neurosurgical diseases. By identifying dysregulated cellular targets or pathways in individual patients, scRNA-seq can inform the development of targeted therapies tailored to the molecular characteristics of a patient’s disease. This approach can lead to more effective and personalised treatments, minimising adverse effects and improving therapeutic responses [69]. For instance, scRNA-seq has been employed to characterise the tumour microenvironment in medulloblastoma, aiding in identifying molecular targets for immunotherapy [70].

##### Therapeutic discovery

scRNA-seq can facilitate the discovery of novel therapeutic targets in neurosurgical diseases by identifying cell populations or specific genes that play crucial roles in disease pathogenesis. This knowledge can guide the development of new therapeutic strategies and the repurposing of existing drugs to target critical pathways or cellular states [71]. For example, scRNA-seq studies in neuroblastoma have revealed distinct molecular subtypes with different drug sensitivities, allowing for the development of tailored treatment approaches [72].

#### Considerations for clinical implementation

##### Standardisation

To ensure the reproducibility and comparability of scRNA-seq data, establishing standardised protocols, quality control measures, and analysis pipelines is essential. Collaboration among researchers and adherence to established guidelines, such as those provided by the Human Cell Atlas project [73], can help promote consistent and reliable results. Furthermore, benchmarking studies comparing different scRNA-seq technologies and computational tools can aid standardisation efforts [74].

##### Sample acquisition and preservation

Adequate sample acquisition and preservation protocols are critical to maintaining the integrity of cellular populations and gene expression profiles in scRNA-seq studies. Optimising tissue processing, storage, and isolation techniques should be considered to minimise technical artefacts and ensure high-quality data. Recent advancements in SC isolation technologies, such as microfluidics-based platforms, have improved the efficiency and reproducibility of sample preparation [70].

##### Scalability and cost-effectiveness

Scalability and cost-effectiveness are essential for the clinical implementation of scRNA-seq. Generating large amounts of data and requiring specialised equipment and expertise poses challenges for widespread adoption. However, technological advances, such as droplet-based scRNA-seq methods, have increased throughput and reduced costs, making scRNA-seq more accessible [75]. Collaboration with clinical laboratories and the development of shared resources can further promote the feasibility of scRNA-seq in clinical settings.

##### Data interpretation and integration

ScRNA-seq data interpretation necessitates expertise in computational biology and the specific neurosurgical context. Integrating scRNA-seq data with other omics data sets, such as genomics or proteomics, can provide a more comprehensive understanding of the molecular mechanisms underlying neurosurgical diseases. Integration strategies, such as trajectory analysis or network-based approaches, enable the identification of key regulatory pathways and potential therapeutic targets [74].

#### Clinical trials and therapeutic validation

The role of scRNA-seq in clinical trials and therapeutic validation is rapidly expanding. ScRNA-seq can assess treatment response, identify predictive biomarkers, and monitor disease progression during clinical trials. For instance, clinical trials leverage scRNA-seq to identify molecular subtypes associated with therapeutic resistance in GBM and guide the development of targeted therapies [76]. In addition, case studies have demonstrated the utility of scRNA-seq in characterising tumour heterogeneity, predicting treatment response, and identifying potential therapeutic targets in various neurosurgical diseases [77, 78].

In one notable study, scRNA-seq was employed to investigate the intricate landscape of paediatric ependymoma, unveiling the intricacies of intratumoral heterogeneity and cellular hierarchy. This analysis unearthed crucial insights into impaired developmental trajectories and potential therapeutic targets [54]. Another study adeptly harnessed scRNA-seq to meticulously elucidate and validate intricate cell–cell communication networks within the tumour microenvironment. This comprehensive mapping provided profound insights into both inter- and intracellular signalling and their profound implications for cancer therapy [79]. In a distinct context, the use of scRNA-seq in studies involving human glioma and immune cells yielded essential information. This technique was instrumental in identifying S100A4 as a promising immunotherapy target, thereby bolstering the development of potential treatment strategies [80]. Furthermore, scRNA-seq analysis conducted on cells derived from both degenerating and non-degenerating intervertebral discs within the same individual ushered in a novel era of biomarker discovery for intervertebral disc degeneration [81].

ScRNA-seq holds immense potential for clinical translation in neurological and neurosurgical research. By providing insights into cellular heterogeneity, identifying biomarkers, and facilitating therapeutic discovery, scRNA-seq can revolutionise clinical practice and improve patient care. However, practical considerations regarding standardisation, sample acquisition, scalability, and data interpretation must be addressed for successful clinical implementation. With ongoing advancements and collaborative efforts, scRNA-seq is poised to transform neurosurgical research and pave the way for personalised treatments. The clinical translation and practical considerations of scRNA-seq in neurological and neurosurgical research is summarised in Table 2.

### Ethical and legal implications

ScRNA-seq has revolutionised the study of complex biology, providing unprecedented insights into heterogeneous cell populations, developmental trajectories, gene transcriptional kinetics, and regulatory networks [1]. While scRNA-seq offers immense potential, it also raises significant ethical concerns that must be carefully addressed.

One of the primary ethical issues associated with scRNA-seq is informed consent. The Belmont Report, which outlines ethical standards for research, needs to be adapted to the evolving field of molecular genetics. As technologies, such as scRNA-seq advance, allowing for the traceability of an individual’s digital genetic fingerprint, determining the extent of initial consent required and the potential future use of samples becomes a critical concern [82]. Data privacy is another ethical concern in scRNA-seq research. With the capacity for unintended discoveries through whole-genome analysis, ensuring individuals’ genomic privacy becomes increasingly crucial. Questions arise regarding who should have access to the data and how existing samples and data from subjects not initially consented for alternate studies should be handled [82]. In addition, potential biases in data interpretation pose an ethical challenge. scRNA-seq generates vast amounts of data, making its interpretation complex. There is a risk of over-interpretation, leading to erroneous conclusions. Therefore, it is vital to employ rigorous statistical methods for data analysis and to validate the results [1].

Responsible data sharing and collaboration play a pivotal role in maximising the scientific and clinical impact of scRNA-seq research. Leveraging large-scale scRNA-seq data through collaboration-driven tools, flexible software environments, and centralised databases can facilitate exploration, annotation, and sharing of results while upholding responsible and ethical data practices [83]. In the context of tumour heterogeneity to axonal regeneration, scRNA-seq holds great promise for investigating tumour cell heterogeneity and identifying therapeutic targets. Furthermore, it offers insights into the molecular mechanisms underlying axonal regeneration, aiding in the discovery of potential therapeutic targets for neurological and neurosurgical disorders. However, it is imperative to address the ethical issues associated with scRNA-seq, including informed consent, data privacy, and potential biases in data interpretation, to ensure responsible and ethical use of the data [1].

### Technical aspects

#### Technical challenges of ScRNA-seq

ScRNA-seq, although revolutionary, confronts an array of technical challenges, particularly when viewed through the lens of neurology and neurosurgery. These technical impediments span the realms of sequencing technologies, data analysis tools, and bioinformatics pipelines, collectively underscoring the complexities and nuances inherent in the neurology-focused applications of scRNA-seq.

##### Data volume and sensitivity

ScRNA-seq, by its very nature, yields a prodigious volume of data, demanding intricate processing and analysis. Furthermore, the choice of sequencing technology exerts a profound influence on the quality and comprehensiveness of the data obtained. For instance, droplet-based approaches, exemplified by 10× Genomics, while widespread, introduce technical noise and may compromise sensitivity when compared to full-length transcript sequencing methods, such as Smart-seq2. This discrepancy poses a considerable challenge, especially in the context of neurology, where capturing rare or low-abundance cell populations can be of paramount importance [84, 85].

##### Analysis of produced suboptimal samples

The effective analysis of scRNA-seq data within the realm of neurology necessitates specialised tools capable of grappling with the high dimensionality and sparsity inherent to this data set. The existing analysis pipelines encounter formidable hurdles, notably the elevated noise rate and heterogeneity encountered within scRNA-seq data. The origin of this experimental noise often traces back to issues, such as diminished RNA capture efficiency, amplification failures during sequencing, and suboptimal sequencing depths. Consequently, transcripts with lower abundances, which may hold vital insights into neural cell function, become challenging to analyse, potentially hampering the depth of our understanding [86, 87].

##### Inefficient bioinformatic pipeline

The challenges of scRNA-seq data analysis are further exacerbated by the need for specialised bioinformatics pipelines uniquely equipped to handle the intricacies of high dimensionality and sparsity. The formidable hurdles of elevated noise and heterogeneity persist in this domain, intimately connected to the broader technical challenges of scRNA-seq. The experimental noise, resulting from factors, such as insufficient RNA capture, amplification issues, and suboptimal sequencing depths, remains a constant spectre, making the analysis of lower abundance transcripts a persistent challenge [88, 89].

#### Overcoming technical challenges; recent advancements, innovations and improvements in ScRNA-seq

ScRNA-seq has witnessed significant technological advancements. Researchers continue to persevere, striving to develop novel methods and tools that promise to surmount its challenges, with the ultimate goal of enhancing the accuracy and reliability of scRNA-seq data analysis in the realm of neurology and neurosurgery. These advancements encompass various aspects, including improvements in sequencing technologies, data analysis tools, and bioinformatics pipelines. The recent innovations have not only expanded our understanding of cellular heterogeneity but have also provided valuable insights into neurological disorders. These technological innovations have the potential to revolutionise the way we approach the study of single-cell genomics and its applications in the field of neuroscience.

i. Advancement in sequencing techniques: Recent advancements in sequencing technologies have substantively bolstered the utility of single-cell RNA sequencing (scRNA-seq). The advent of refined platforms and methodologies, notably droplet-based techniques and Smart-seq, has overcome challenges associated with data sparsity, consequently enhancing detection efficacy [90]. Furthermore, these technological improvements shine a light on more in-depth analysis of brain cell types and gene expression patterns, which is essential for understanding complex neurological conditions [91].
ii. Improving and innovating analytic tools: The need for higher processing capacity for larger volumes of data through scRNA-seq has led to the emergence of specialised data analysis tools capable of managing the large amount of information these techniques yield. Notable computational methodologies, such as Seurat and SingleR, have become instrumental in providing information on the cellular heterogeneity within neurological tissues [92]. These tools offer greater insights into cellular dynamics and functionality. For instance, an analysis of scRNA-seq data has enabled the delineation of distinct cell populations within the hippocampus, thereby enriching the understanding of its intricate cellular landscape [93].
iii. Improving bioinformatics pipelines: Advanced bioinformatics pipelines, tailored for scRNA-seq data analysis, have been instrumental in providing pivotal insights from neuronal cell samples, thereby enriching our understanding of various NDs and other neurological diseases. Efforts are being undertaken to improving the accuracy and efficiency of these pipelines, ensuring the robust analysis of scRNA-seq data [94]. In addition, the development of end-to-end pipelines such as scRNASequest has simplified the process of scRNA-seq data analysis, making it more accessible and efficient [95].

### Future prospects

The rapid progress of ScRNA-seq technology has provided unprecedented opportunities to gain insights into the intricate cellular heterogeneity present in neurological and neurosurgical diseases. By enabling the profiling of individual cells at a transcriptomic level, this revolutionary approach has transformed our understanding of cellular diversity and dynamics. As a result, ScRNA-seq holds significant promise for numerous potential applications within neurological and neurosurgical research.

#### Prospects of ScRNA-seq in neurological and neurosurgical research: cell type classification, disease mechanisms, and spatial transcriptomics

##### Cell type classification and subtyping

The integration of scRNA-seq data with advanced computational algorithms enables the precise identification and classification of distinct cell types in the central nervous system. Langlieb et al. [96] employed scRNA-seq to identify over 100 cell types in the mouse brain, thereby providing a comprehensive atlas of neural cell diversity. Similarly, Lake et al. [97] characterised cell types across the human brain and discovered previously unknown subtypes of inhibitory neurons. These studies illustrate the capability of scRNA-seq to refine existing cell taxonomies and reveal novel cellular subpopulations.

##### Disease mechanisms and pathways

scRNA-seq provides a unique opportunity to elucidate disease mechanisms and pathways by examining gene expression changes at the single-cell level. By comparing transcriptomic profiles of cells from healthy and diseased tissues, researchers can identify disease-associated gene signatures and uncover novel regulatory networks. Luquez et al. [98], for instance, used scRNA-seq to identify transcriptional changes associated with Alzheimer’s disease (AD), revealing alterations in gene expression within specific cell types involved in neuronal function and immune response. Similarly, Couturier et al. [10] applied scRNA-seq to glioblastoma samples, revealing cellular heterogeneity and gene expression patterns associated with disease progression. These studies exemplify the valuable insights scRNA-seq can provide into disease mechanisms, facilitating the development of targeted therapeutic strategies.

##### Spatial transcriptomics

The integration of scRNA-seq with spatial transcriptomic techniques allows for the examination of gene expression patterns within tissue architecture. This emerging field provides valuable insights into the influence of cell-to-cell interactions, cellular organisation, and spatial relationships on disease progression. Jain et al. [17], for example, combined scRNA-seq with spatial transcriptomics to investigate the spatial distribution of cell types and gene expression patterns in human glioblastoma (GBM). Their findings revealed distinct tumour microenvironments and provided insights into the cellular interactions driving tumour growth. Similarly, Vanrobaey et al. [99] employed spatial transcriptomics to map gene expression patterns in the mouse brain, uncovering regional differences in gene expression and cellular composition. These studies highlight the potential of spatial transcriptomics to offer a comprehensive understanding of the spatial heterogeneity underlying neurological and neurosurgical diseases.

#### The potential of scRNA-seq in neurological and neurosurgical disease: diagnosis, prognosis, and treatment

##### Enhancing diagnostic accuracy

The application of scRNA-seq in clinical settings has the potential to revolutionise neurological and neurosurgical diagnostics. By characterising disease-specific transcriptional profiles, scRNA-seq can be a powerful tool for accurate disease classification and subtyping. For instance, Castro et al. [100] utilised scRNA-seq to classify and identify novel subtypes of paediatric brain tumours, providing insights into tumour biology and potential therapeutic targets. Similarly, Sathyamurthy et al. [101] employed scRNA-seq to classify cell types in the human spinal cord, enabling the identification of new markers for spinal cord injuries. These studies demonstrate how scRNA-seq can complement traditional diagnostic approaches and provide clinicians with more precise and reliable information for personalised treatment decisions.

##### Improving prognostic evaluation

The ability to capture the heterogeneity within neurological and neurosurgical diseases through scRNA-seq can contribute to a more accurate prognostic evaluation. By identifying specific cellular subsets associated with disease progression or treatment response, scRNA-seq can assist in predicting clinical outcomes. For instance, Wang et al. [102] employed scRNA-seq to analyse glioblastoma samples and identified distinct tumour cell subpopulations associated with patient survival. Their findings underscored the prognostic value of cellular heterogeneity in glioblastoma and its potential for guiding personalised treatment strategies. Similarly, Hook et al. [103] employed scRNA-seq to characterise the heterogeneity of dopaminergic neurons associated with Parkinson’s disease, revealing specific subpopulations with differential vulnerability to degeneration. These studies highlight how scRNA-seq can provide valuable prognostic insights to guide treatment decisions and enhance patient outcomes.

##### Targeting therapeutic approaches

scRNA-seq has the potential to transform therapeutic strategies in neurological and neurosurgical research. By identifying specific cell types or subpopulations driving disease pathogenesis, researchers can develop targeted therapies to modulate disease-specific gene expression profiles. For example, Liu et al. [104] employed scRNA-seq to identify disease-associated cell types in amyotrophic lateral sclerosis (ALS), facilitating the development of potential therapeutic targets for this devastating disease. In addition, Garofano et al. [105] used scRNA-seq to uncover distinct subtypes of glioblastoma stem-like cells, revealing vulnerabilities that could be targeted with specific drugs. These studies exemplify how scRNA-seq can aid in identifying novel therapeutic targets and developing personalised treatment options for patients.

## Conclusion

ScRNA-seq has emerged as a powerful tool that has revolutionised our understanding of neurological disorders, providing high-resolution insights into cellular heterogeneity and gene expression profiles. Its application in neurological tumours, neurodegenerative diseases, and epileptic disorders has resulted in the discovery of novel cell types, disease-specific gene expression changes, and potential therapeutic targets. While scRNA-seq presents certain challenges and limitations, its potential for translation into clinical medicine is promising, paving the way for precision approaches in neurological and neurosurgical research and practice. By unravelling the intricate cellular landscape and molecular mechanisms underlying these disorders, scRNA-seq provides a valuable resource for further investigations and the development of targeted therapeutic interventions. As this field continues to evolve, it is expected that scRNA-seq will play an increasingly important role in shaping the future of neurological research and improving patient outcomes.

## Appendix

See Tables 1, 2.

**Table 1:** Summary of methodology for this review

Methodology steps	Description
Literature search	PubMed, EMBASE, Google Scholar, the Cochrane Library, and Scopus
Inclusion criteria	Full-text articles published in English Publication date range: 2003–2023 (past two decades) Focus on neurosurgery and neurology
Exclusion criteria	Standalone abstracts Case reports Posters Unpublished or non-peer-reviewed studies
Search terms	Keywords such as “scRNA Sequencing” and “Single-cell sequencing” coupled with indicators, such as “Brain Tumours”, “Spinal Cord Tumours”, “Neurodegenerative Disorders”, “Cerebrovascular Disorders”, “strokes”, and “epilepsy”
Additional search	Manual examination of references cited in recent disease-specific reviews No predetermined limit on the number of studies Encompassing diverse study designs: • Descriptive studies • Animal-model studies • Cohort studies • Observational studies Including investigations in both pre-clinical and clinical settings

**Table 2:** Clinical translation and practical considerations of single-cell RNA sequencing in neurological and neurosurgical research

Research studies	Key points
Translational potential of scRNA-seq [68, 69, 71]	ScRNA-seq enables the identification of biomarkers for neurosurgical diseases Facilitates precision medicine and personalised therapies Contributes to therapeutic discovery
Considerations for clinical implementation [70, 74, 75]	Standardisation of protocols, quality control, and analysis pipelines Importance of sample acquisition and preservation Scalability and cost-effectiveness Data interpretation and integration
Clinical trials and therapeutic validation [76]	scRNA-seq aids in treatment response assessment and identification of predictive biomarkers Monitoring disease progression during clinical trials

*scRNA-seq* single-cell ribonucleic acid sequencing

## Data Availability

Not applicable.

## Abbreviations

scRNA-seq: Single-cell ribonucleic acid sequencing
RNA: Ribonucleic acid
Single-Cell RNA: ScRNA
SC: Single-cell
UMI: Unique molecular identifiers
mRNA: Messenger ribonucleic acid
DNA: Deoxyribonucleic acid
cDNA: Complementary deoxyribonucleic acid
NGS: Next generation sequencing
VEGF-A: Vascular endothelial growth factor-A
GBM: Glioblastoma multiforme
snRNA-seq: Single-nuclei ribonucleic acid sequencing
PCR: Polymerase chain reaction
ND: Neurodegenerative diseases
CIN: Chromosomal instability
CSC: Cancer stem cells
CAF: Cancer-associated fibroblasts
SULT1E1: Sulfotransferase family 1E member 1
MO: Meningioma organoid
NF2: Neurofibromin 2
HLA: Human leukocyte antigen
CDH2: Cadherin 2
ADC: Anti-drug conjugate
AD: Alzheimer’s disease
PD: Parkinson’s disease
ALS: Amyotrophic lateral sclerosis
HD: Huntington’s disease
MS: Multiple sclerosis
CRISPR: Clustered regularly interspaced short palindromic repeats
PTPRZ1: Receptor-type tyrosine-protein phosphatase zeta
iPSC: Induced pluripotent stem cell
CADPS2: Calcium-dependent secretion activator 2
GFAP: Glial fibrillary acidic protein
TH: Tyrosine hydroxylase
TLE: Temporal lobe epilepsy
OPC: Oligodendrocyte precursor cells
OLIG2: Oligodendrocyte transcription factor 2
DRE: Drug-refractory epilepsy
IL1B: Interleukin-1 beta
IGSF3: Immunoglobulin superfamily member 3
Kir4.1: Inwardly rectifying potassium channel
PBMC: Peripheral blood mononuclear cells
PC: Poorly controlled
WC: Well controlled
IL-1: Interleukin 1
SUDEP: Sudden unexpected death in epilepsy
SCI: Spinal cord injuries
SPP1: Secreted phosphoprotein 1
DAM: Disease-associated microglia
IGF1: Insulin-like growth factor 1
NSCs: Neural stem cells
TAM: Tumour associated macrophage
ATAC-seq: Assay for transposase-accessible chromatin using sequencing
Tregs: Regulatory T cells
TGF-β: Transforming growth factor beta
ICH: Intracranial haemorrhage
